# Visualization of hypoxia in cancer cells from effusions in animals and cancer patients

**DOI:** 10.3389/fonc.2022.1019360

**Published:** 2022-12-22

**Authors:** Yue Li, Long Zhao, Yunlong Huo, Xianghong Yang, Yong Li, Hao Xu, Xiao-Feng Li

**Affiliations:** ^1^ Department of Nuclear Medicine, The Second Clinical Medical College, Jinan University (Shenzhen People’s Hospital), Shenzhen, China; ^2^ The First Affiliated Hospital, Jinan University, Guangzhou, China; ^3^ Department of Nuclear Medicine, The First Affiliated Hospital, Southern University of Science and Technology, Shenzhen, China; ^4^ Department of Pathology, Shengjing Hospital of China Medical University, Shenyang, China; ^5^ Department of Nuclear Medicine, Shenzhen Hospital of Southern Medical University, Bao’an, Shenzhen, China; ^6^ Department of Nuclear Medicine, the First Affiliated Hospital, Jinan University, Guangzhou, China; ^7^ Department of Medical Physics, Memorial Sloan-Kettering Cancer Center, New York, NY, United States

**Keywords:** hypoxia, proliferation, ascites, pleural effusion, cancer cells, chemo-radiotherapy resistance, cancer patients

## Abstract

**Objective:**

Tumor hypoxia is frequently observed in primary solid malignancies, but the hypoxic status of tumor cells floating in body cavity effusions is largely unknown, especially in patients. This study was to observe the hypoxia and proliferation status of cancer cells floating in effusions in mice and patients.

**Methods:**

The distribution of hypoxia in cancer cells floating in ascites was first studied in nude mice. Hypoxia was detected by immunofluorescent visualization of pimonidazole and GLUT-1. For cancer patients, we retrospectively collected 21 ascites and 7 pleural effusion sample blocks of cancer patients, which were confirmed to contain tumor cells. Immunohistochemistry was performed to detect the expression of endogenous hypoxic markers HIF-1α and GLUT-1, proliferation index Ki-67. ^18^F-FDG PET/CT was performed to detect the glucose metabolism status of tumor cells in effusions.

**Results:**

The tumor cells collected from ascites were positive for pimonidazole and GLUT-1, which suggesting that the cancer cells floating in ascites were hypoxic. Patterns of tumor hypoxia in human patients are similar to those observed in animal. HIF-1α and GLUT-1 were expressed by tumor cells in nearly all 28 cytological cases. For Ki-67 index, ascites tumor cells had a relatively low expression level compared with their corresponding primary or its metastatic lesions. Tumor cells in effusions showed high ^18^F-FDG uptake indicated the enhanced activity of glucose metabolism.

**Conclusion:**

Tumor cells in body cavity effusions, as a unique subgroup of tumor, are in a state of hypoxia and low proliferation, which would be one of the driven causes of chemo-radiotherapy resistance. Novel therapeutic interventions are urgently needed to overcome tumor hypoxia.

## 1 Introduction

Malignant body cavity effusion, mainly ascites and pleural effusion, is a prominent feature of advanced cancer which carries a shorter survival and lower quality of life. Malignant ascites is most common in ovarian cancer (37%), followed by liver-biliary and pancreatic tumors (21%) and gastrointestinal cancer (18%). Malignant pleural effusion commonly originates from lung cancer (37.5%), breast cancer (16.8%) and lymphoma (11.5%) (1). The median survival is only approximately 5.7 months (2). Cytoreductive surgery followed by chemotherapy is the current first-line treatment which has lasted nearly two decades. Although about 80% of the patients will initially sensitive, most of them will recur and ultimately become chemotherapy resistant (3). In recent years, many new therapeutic schemes have been proposed (3, 4), including hyperthermic intra-peritoneal chemotherapy (HIPEC) (5–8), molecular targeted therapy (9, 10), immunotherapy (11) and so on, and encouraging results have been reported. However, the current reported 5-year survival rate of these cancers has no significant improvement in the past 25 years (12, 13). Therefore, in order to seek more effective treatment strategies, the pathophysiological and biological characteristics of malignant body cavity effusions still need to be further investigated.

Although the existence of tumor hypoxia is a common feature of primary solid malignancies (14, 15). Our previous pilot studies in animals have showed that peritoneal disseminated submillimeter micro-metastases of colorectal carcinoma are severely hypoxic, and the cells were in a state of low proliferation (16–20). However, the hypoxic status of tumor cells floating in ascites is largely unknown. It is well known that the cytotoxic effectiveness of many chemotherapeutic agents is decreased by hypoxia (21–23). The commonly used radiotherapy and most chemotherapy only act on the cells in mitotic phases by interfering with its DNA synthesis. The proportion of proliferating cancer cells in tumors determines the effect of traditional chemo-radiotherapy to a large extent (24, 25). It thus follows that, if tumor cells floating in ascites were hypoxic, it may be relatively resistant to traditional chemo-radiotherapy.

However, there is no relevant study on the hypoxic state of tumor cells in body cavity effusions. Are tumor cells floating in effusions in a state of hypoxia, low proliferation that similar to the results of peritoneal disseminated micro-metastases? Is the hypoxic state of human effusion tumor cells consistent with the results of animal experiments? That is what this study attempts to reveal, and is also the originality of this study.

In animal experiments, it is possible to observe tumor hypoxia using a variety of means. We used exogenous hypoxia marker injection and imaging (immunofluorescence and autoradiography) to detect tumor hypoxia. Pimonidazole is a 2-nitroimidazole compound that is selectively reduced and binds to intracellular macromolecules in hypoxic regions of tumors. It has been widely used in animal experimental studies of tumor hypoxia. Autoradiography using the glucose analog ^18^F-FDG can reflect the glucose metabolism level of tumor tissue and indirectly indicate the hypoxia state. While in retrospective human specimen, those methods are not applicable. We and others have compared and validated the co-localization of some exogenous hypoxic markers such (Pimonidazole, EF5) and endogenous hypoxic markers such as hypoxia factor hypoxia-inducible factor-1 alpha (HIF-1α) and Glucose Transporter-1 (GLUT-1). In this study, we used the method of immunohistochemical staining of endogenous hypoxia factor HIF-1α and GLUT-1, and proliferation marker Ki-67. HIF-1α is the transcription factor modulating many of the hypoxia related genes and plays an essential role in cellular oxygen homeostasis. Under normoxic condition, HIF-1α is located in the cytoplasm and has a short half-life, while in hypoxic condition, stabilized HIF-1α translocates to the nucleus (26, 27). So nuclear staining of HIF-1α indicates the presence of hypoxia. Ki-67 is an important proliferation marker that widely used clinically. It is a nuclear protein detected in proliferating human cells, while quiescent (G0 phase) cells were negative for the Ki-67 antigen (28).

In this report, the hypoxia and proliferation status of tumor cells floating in effusions, mainly ascites, was detected in both tumor bearing mice and cancer patients, aiming at point out a new direction for the clinical treatment of tumors.

## 2 Materials and methods

We conduct double verification through animal experiments and human specimen research.

### 2.1 Animal experiments

#### 2.1.1 Tumor cell lines and animals

Cell lines: human tumor cell line used in experiments is human colorectal adenocarcinoma HT29 and human non-small cell lung cancer HTB177, which is all purchased from American Type Cell Collection (Manassas, VA). Cells were maintained in McCoy’s 5A modified medium (Gibco, Grand Island, NY), and grown at 37°C in a humidified CO_2_ incubator. All media were supplemented with 10% fetal bovine serum (Gemini, West Sacramento, CA), 1% glutamine and 1% antibiotic mixture (Cellgro). Exponentially growing cells were harvested with 0.05% trypsin plus EDTA, washed and suspended in phosphate buffered saline (PBS).

Animals: 6-8 week old female athymic NCr-*nu/nu* mice purchased from NCI-Frederick Cancer Research Institute (Bethesda, MD) were used. The experimental protocols were approved by the Institutional Animal Ethics Committee.

#### 2.1.2 Establishment of tumors in animals

Ascites tumors were induced by injecting tumor cell suspensions (5-10×10^6^ cells/0.1-0.2ml) into the peritoneal cavity. Five mice for each cell line. Animals were sacrificed typically 6-7 weeks after tumor initiation, when ascites was evident in the majority of animals. The ascites fluid was observed to be bloody and contained a distribution of free-floating tumor cell aggregates of sizes up to 1 mm in diameter.

#### 2.1.3 Exogenous markers of tumor hypoxia

The hypoxia cell marker pimonidazole hydrochloride (20 mg/ml in physiological saline; 80mg/kg; nominal injected volume 0.1 ml) and ^18^F-FDG (~7.4 MBq, 0.1ml) were administered *via* tail vein injection 1 hr before animal sacrifice.

#### 2.1.4 Preparation of frozen tumor sections

Immediately after animal sacrifice, ascites fluid were removed for subsequent processing. Ascites tumors were harvested, washed with ice cold PBS to remove red blood cells, frozen and embedded in optimal-cutting-temperature medium (4583; Sakura Finetek). For all tumor samples, sets of 10 contiguous 8μm thick tissue sections were cut using a Microm HM500 cryostat microtome (Microm International GmbH, Walldorf, Germany) and adhered to poly-L-lysine coated glass microscope slides. Frozen sections were stored at -80°C until use.

#### 2.1.5 Visualization of hypoxia on tumor sections

Slides were air-dried, fixed in cold acetone (4°C) for 20 min, and incubated with SuperBlock (Pierce Biotechnology, Rockford, IL) at room temperature for 30 min. Sections were then incubated with FITC conjugated anti-pimonidazole monoclonal antibody (Chemicon International), diluted 1:25, for 1 hr at room temperature. GLUT-1 staining was performed on the adjacent section by incubating for 1 hr at room temperature with rabbit anti-GLUT-1 polyclonal antibody (Millipore) diluted 1:50. Sections were washed three times in PBS, each wash lasting 5 min, and incubated for 1 hr at room temperature with AlexaFluor488-conjugated goat antirabbit anti-body (1:100, Molecular Probes) and washed again. To control for nonspecific binding of antibodies, stained sections were processed from similar tumors that had not been exposed to pimonidazole. Controls for GLUT-1 staining consisted of sections in which primary antibody was omitted.

Images were acquired at ×100 magnification using an Olympus BX40 fluorescence microscope (Olympus America Inc., Melville, NY) equipped with a motorized stage (Prior Scientific Instruments Ltd., Cambridge, UK). Pimonidazole was imaged using green filter while GLUT-1 was imaged using red filter.

After acquisition of fluorescence images, the same tumor sections were stained with hematoxylin and eosin (H&E) and imaged by light microscopy. Microscopic images were coregistered and analyzed using Photoshop 8.0 (Adobe).

#### 2.1.6 ^18^F-FDG DAR

As described previously (18), autoradiograms were obtained by placing the tumor sections in a film cassette against a Fujifilm BAS-MS2325 imaging plate (Fuji Photo Film Co.). Plates were exposed overnight and read by a Fujifilm BAS-1800II bioimaging analyzer (Fuji Photo Film Co.), which generated digital images with the pixel dimensions is 50 µm x 50 µm, and the single pixel dimension is 50 µm x 50 µm x 8 µm.

### 2.2 Human specimen research

#### 2.2.1 Human cytological specimen acquisition

We retrospectively reviewed all the cytological cases of body cavity effusions, mainly ascites and pleural effusions, from January to September 2020 in Shengjing Hospital of China Medical University, Shenyang, China. Samples of 28 patients were selected, whose age range from 19~ 77 years old, 4 male patients and 24 female patients. All the samples had been already made into cell blocks and had been diagnosed as ascites and pleural effusion cancer through immunohistochemistry and their corresponding histological specimen of primary and/or metastatic lesions were also collected if available. Tumor types include 15 cases of ovarian carcinoma, 6 cases of lung adenocarcinoma, 3 cases of gastric adenocarcinoma, 2 cases of colorectal adenocarcinoma, 1 case of breast carcinoma and 1 case of hilar cholangiocarcinoma. The detailed information is shown in **Table 1**.

**Table 1:** Cases of malignant body cavity effusions.

No.	Age	Sex	Primary site	Tumor type
1	60	Female	Ovary	High-grade serous carcinoma
2	56	Female	Ovary	High-grade serous carcinoma
3	61	Female	Ovary	High-grade serous carcinoma
4	60	Female	Ovary	Clear cell carcinoma
5	55	Female	Ovary	Clear cell carcinoma
6	57	Female	Ovary	High-grade serous carcinoma
7	62	Female	Ovary	High-grade serous carcinoma
8	51	Female	Ovary	High-grade serous carcinoma with a small amount of clear cell carcinoma
9	27	Female	Ovary	High-grade serous carcinoma
10	59	Female	Ovary	High-grade serous carcinoma
11	47	Female	Ovary	High-grade serous carcinoma
12	42	Female	Ovary	High-grade serous carcinoma
13	77	Female	Ovary	High-grade serous carcinoma
14	46	Female	Ovary	High-grade serous carcinoma
15	27	Female	Ovary	High-grade serous carcinoma
16	57	Female	Stomach	Adenocarcinoma
17	59	Male	Stomach	Adenocarcinoma
18	34	Female	Stomach	Adenocarcinoma
19	19	Female	Colon	Adenocarcinoma
20	66	Male	Colon	Adenocarcinoma
21	57	Female	Liver	Cholangiocarcinoma
22	67	Female	Lung	Adenocarcinoma
23	72	Female	Lung	Adenocarcinoma
24	71	Male	Lung	Adenocarcinoma
25	68	Male	Lung	Adenocarcinoma
26	63	Female	Lung	Adenocarcinoma
27	68	Female	Lung	Adenocarcinoma
28	72	Female	Breast	Breast carcinoma

The Ethics Committee of Shengjing Hospital of China Medical University approved the study.

#### 2.2.2 Immunohistochemistry

The blocks of selected cases were then sectioned continuously at 4 μm and were placed in a 60°C oven for more than 3 hrs. Hematoxylin and Eosin (H&E) staining was conducted first using a Tissue-Tek Prisma slide stainer (Sakura Seiki, Japan).

The sections were initially dewaxed through xylene (four times, 5 min each time), and then gradient ethanol (100% for 5 min, 100% for 5 min, 95% for 3 min, 80% for 2 min respectively) then into water using Tissue-Tek Prisma slide stainer (Sakura Seiki, Japan). After washing with distilled water, the dewaxed sections were incubated in Citrate buffer pH 6.0 (Abcam, UK) and performed high pressure heat-induced antigen retrieval for 3 min. After cooled to room temperature, they were rinsed with PBS 3 times, 3 min each time. Then, the sections were incubated in peroxidase inhibitors for 10 min at room temperature to block the activity of endogenous peroxidases, and then rinsed with PBS 3 times, 3 min each time. The primary antibody HIF-1α (diluted 1:500, Abcam, UK), GLUT-1 (diluted 1:2000, Abcam, UK), Ki-67 (diluted 1:1000, Abcam, UK) and CD31 (diluted 1:1000, Abcam, UK) was added (for negative control, only PBS buffer was added) and the slides were placed in a humid chamber at 4°C overnight. After rewarming to room temperature and rinsing with PBS 3 times, 3 min each time, secondary antibody (Goat Anti-Rabbit IgG H&L, Abcam, UK) was added, followed by incubation for 20 min at room temperature and then rinsed with PBS 3 times, 3 min each time. Freshly prepared diaminobenzidine (DAB) solution was added. The sections were then observed under a Leica DM2000 microscope at ×100 magnification (Leica, Germany) until desired color is achieved and then rinsed with running water for 5 min. Hematoxylin was used to stain the nucleus for 3 min at room temperature and then the sections were rinsed with running water for 5 min followed by dehydration of pure alcohol for 5 min. Finally, the sections were sealed with neutral gum.

#### 2.2.3 Image processing

All the sections were scanned by the NanoZoomer Digital Pathology (Hamamatsu, Japan) and stored for further image analysis. The fields of interest were exported at ×200 or ×400 magnification.

#### 2.2.4 Evaluation of immunohistochemical results

HIF-1α and Ki-67 positive was marked by the presence of yellow or brown staining in cell nuclei. While GLUT-1 positive was marked by the presence of yellow or brown staining in cell membrane. Positive tumor cells of HIF-1α, GLUT-1 and Ki-67 were counted at ×400 magnification and the results were expressed as the percentage of positive cells per 100 tumor cells (Randomly select 5 high power fields and take the average).

#### 2.2.5 PET/CT imaging

After 4-6 hours of fasting, all patients underwent an intravenous injection of ^18^F-FDG at a dose of 5.5 MBq/kg. Following a rest for 1 hour, PET/CT scan of the head and trunk was performed using a combined PET/CT biograph (GE Healthcare, United States). All scans were performed in a three-dimensional model. After attenuation correction and iterative reconstruction, the slice thickness was 3.75 mm, and the in-plane resolution of this system was 3.27 mm.

#### 2.2.6 PET image analysis

All the images were retrospectively read by two experienced nuclear medicine physicians. Hypermetabolism was defined as FDG activity higher than that of adjacent normal soft-tissue structures. The maximum standardized uptake value (SUVmax) of tumors and effusions were measured.

#### 2.2.7 Statistical analysis

Data were analyzed with SPSS22.0. Statistical significance was examined by t test. A *P* value less than 0.05 was considered a statistically significant difference.

## 3 Results

### 3.1 Visualization of hypoxia in ascites tumors of tumor bearing mice by immunofluorescent

**Figure 1:**
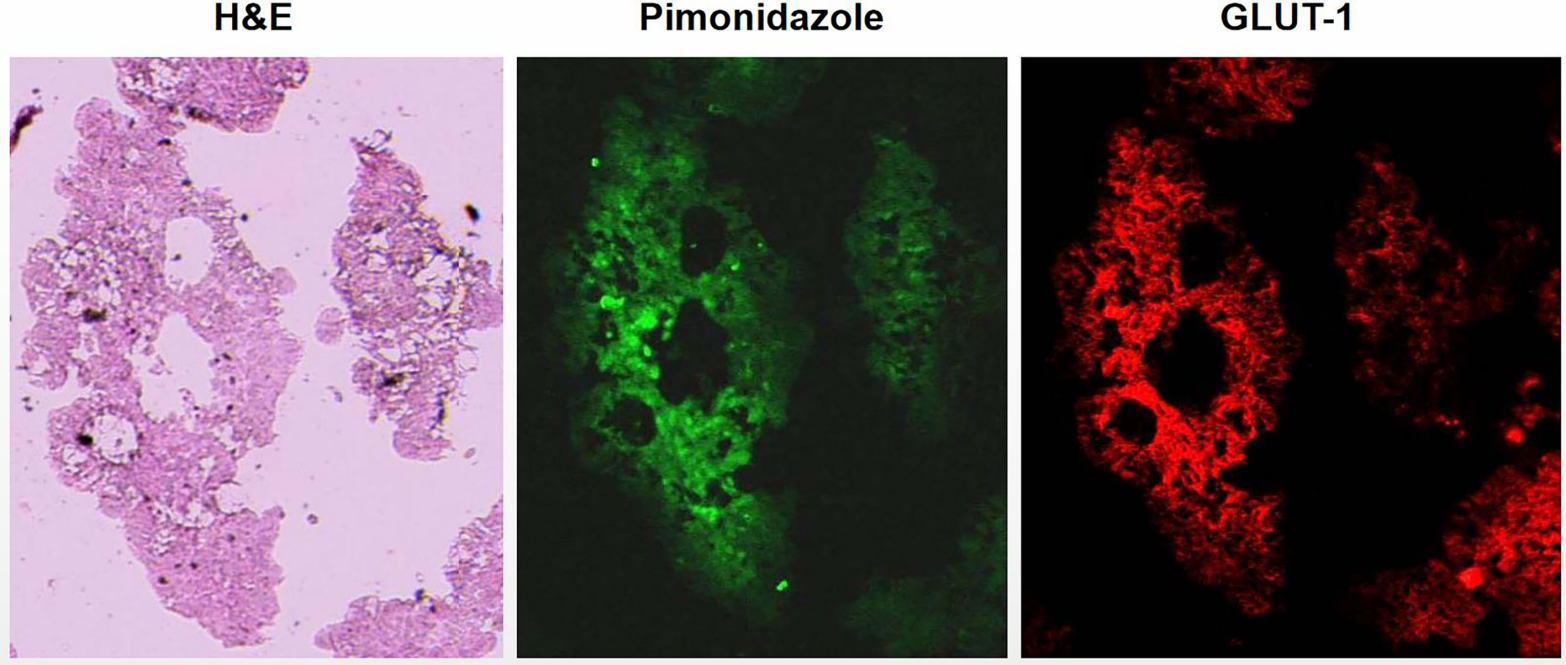
Visualization of hypoxia in ascites tumors of tumor bearing mice by immunofluorescent. Ascites tumor cells of HT29 cell line. Tumor cells were strongly positive for hypoxia markers pimonidazole and GLUT-1 indicated that ascites tumor cells were hypoxic.

**Figure 2:**
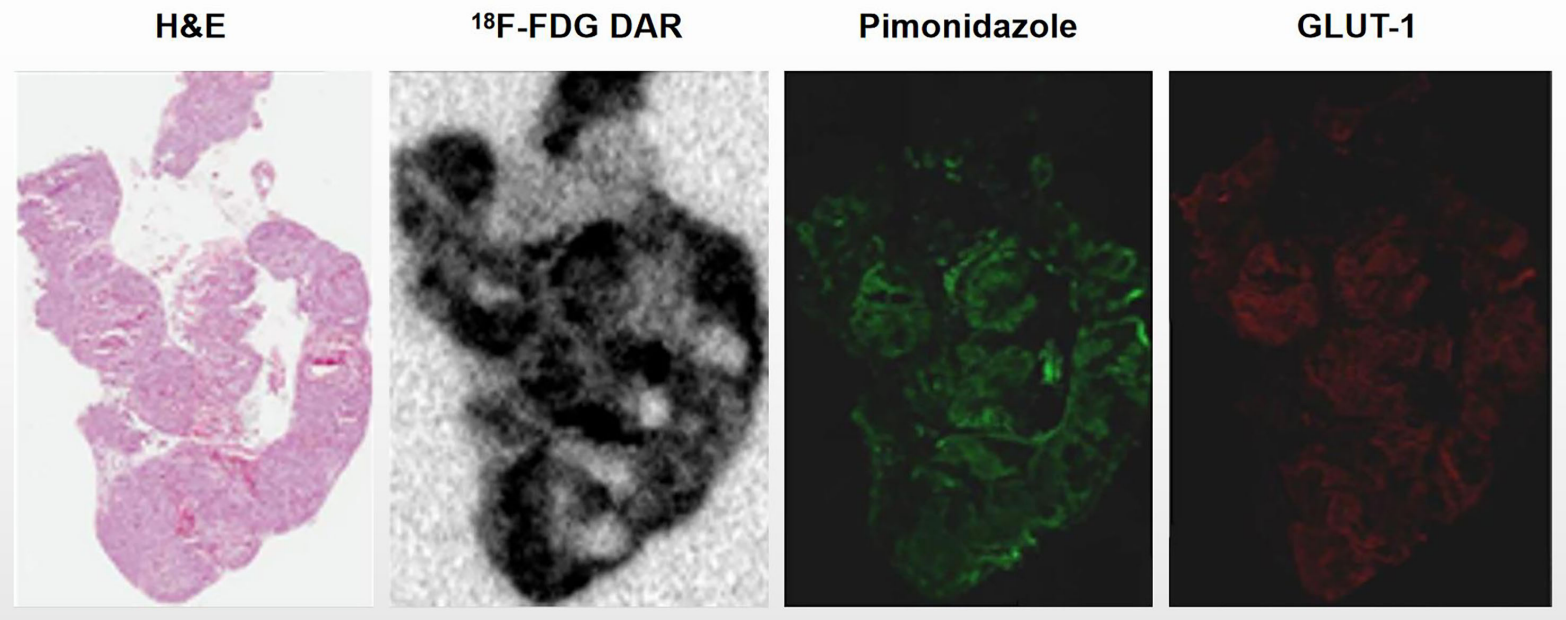
Ascites tumors showed high ^18^F-FDG accumulation. Ascites tumor cells of HTB177 cell line. Tumor cells showed high uptake of ^18^F-FDG by DAR, suggesting that their glucose metabolism activity was increased; Meanwhile, hypoxia markers pimonidazole and GLUT-1 were also highly expressed.

Ascites tumors showed intensely positive for pimonidazole and GLUT-1, indicating that they were mostly hypoxic (**Figure 1**). High uptake of ^18^F-FDG was observed by DAR, suggesting that the glucose metabolism of tumor cells was enhanced, which was also a manifestation of hypoxia (**Figure 2**).

### 3.2 Visualization of hypoxia in ascites tumors of cancer patients by immunohistochemistry

**Figure 3:**
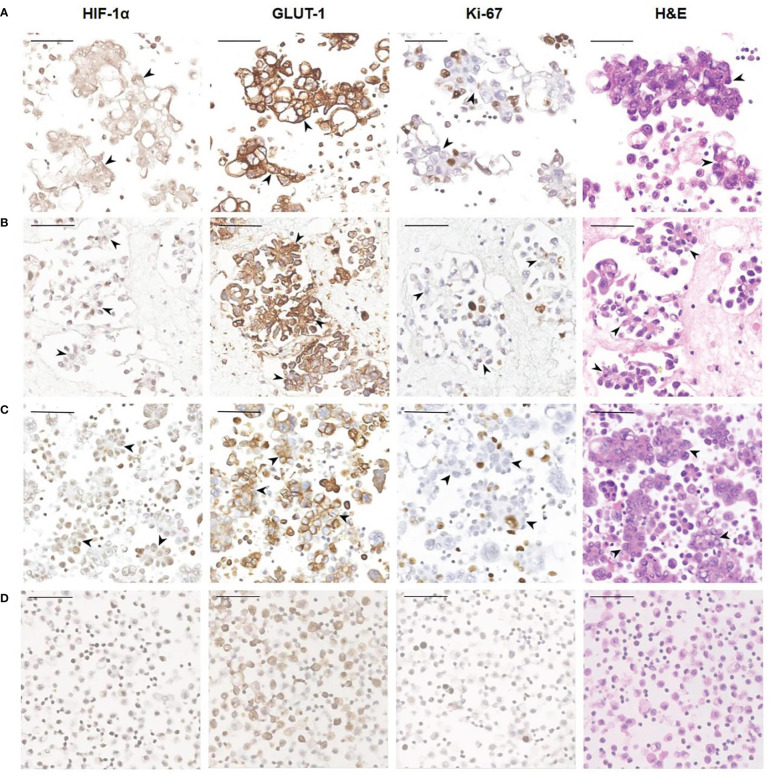
Representative images showing HIF-1α, GLUT-1 and Ki-67 expression of human ovarian cancer cells in ascites. **(A)** A 56-year-old female diagnosed with high-grade serous carcinoma of the ovary. The black arrowheads show clusters of tumor cells in ascites (400×). These tumor cells are HIF-1α positive and GLUT-1 positive with a relatively low Ki-67 index. *Bar*, 50 μm. **(B)** A 61-year-old female was diagnosed with high-grade serous carcinoma of the ovary. The black arrowheads show the papillary arrangement of tumor cells in ascites (400×). These tumor cells are HIF-1α positive and GLUT-1 positive with a relatively low Ki-67 index. *Bar*, 50 μm. **(C)** A 57-year-old woman diagnosed with high-grade serous carcinoma of the ovary. The black arrowheads show the papillary arrangement of tumor cells in ascites (400×). These tumor cells are HIF-1α positive, GLUT-1 positive with a relatively low Ki-67 index. *Bar*, 50 μm. **(D)** Non-neoplastic benign effusion. There are inflammatory cells mainly composed of lymphocytes and reactive mesothelial cells. Some of these cells also express HIF-1α and GLUT-1, and the Ki-67 index is low. *Bar*, 50 μm.

**Figure 4:**
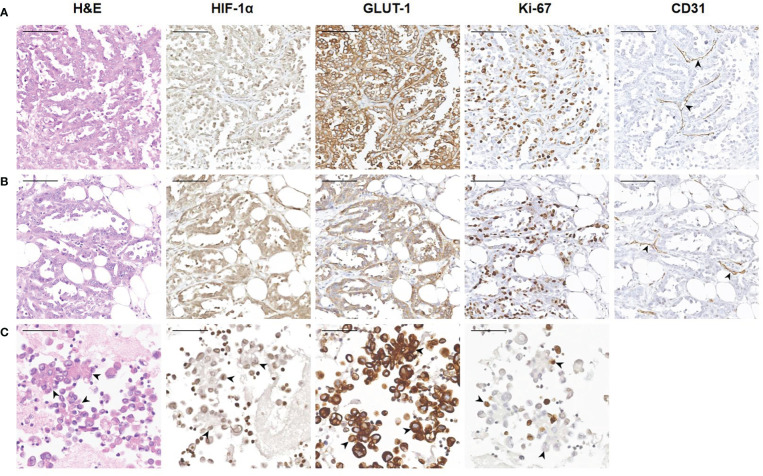
Representative images showing HIF-1α, GLUT-1 and Ki-67 expression of human ovarian cancer between tumor cells in ascites with corresponding primary and peritoneal lesions. A 60-year-old woman was diagnosed with high-grade serous carcinoma of the ovary. **(A)** H&E and immunohistochemical staining show the tumor cells in the ovary (200×). The tumor cells are arranged in a papillary or glandular pattern, and are HIF-1α positive and GLUT-1 positive with a high Ki-67 index. Through CD31 staining, several blood vessels are identified in the stroma (black arrowheads). *Bar*, 100 μm. **(B)** Immunohistochemical results of tumor cells in a peritoneal metastasis (200×). Tumor cells infiltrate the adipose tissue, and are also HIF-1α positive and GLUT-1 positive with a high Ki-67 index. Blood vessels are visible (black arrowheads). *Bar*, 100 μm. **(C)** Immunohistochemical results of tumor cells in ascites (400×). The black arrowheads show the papillary arrangement of tumor cells. These tumor cells are HIF-1α positive and GLUT-1 positive with a relatively low Ki-67 index. *Bar*, 50 μm.

**Figure 5:**
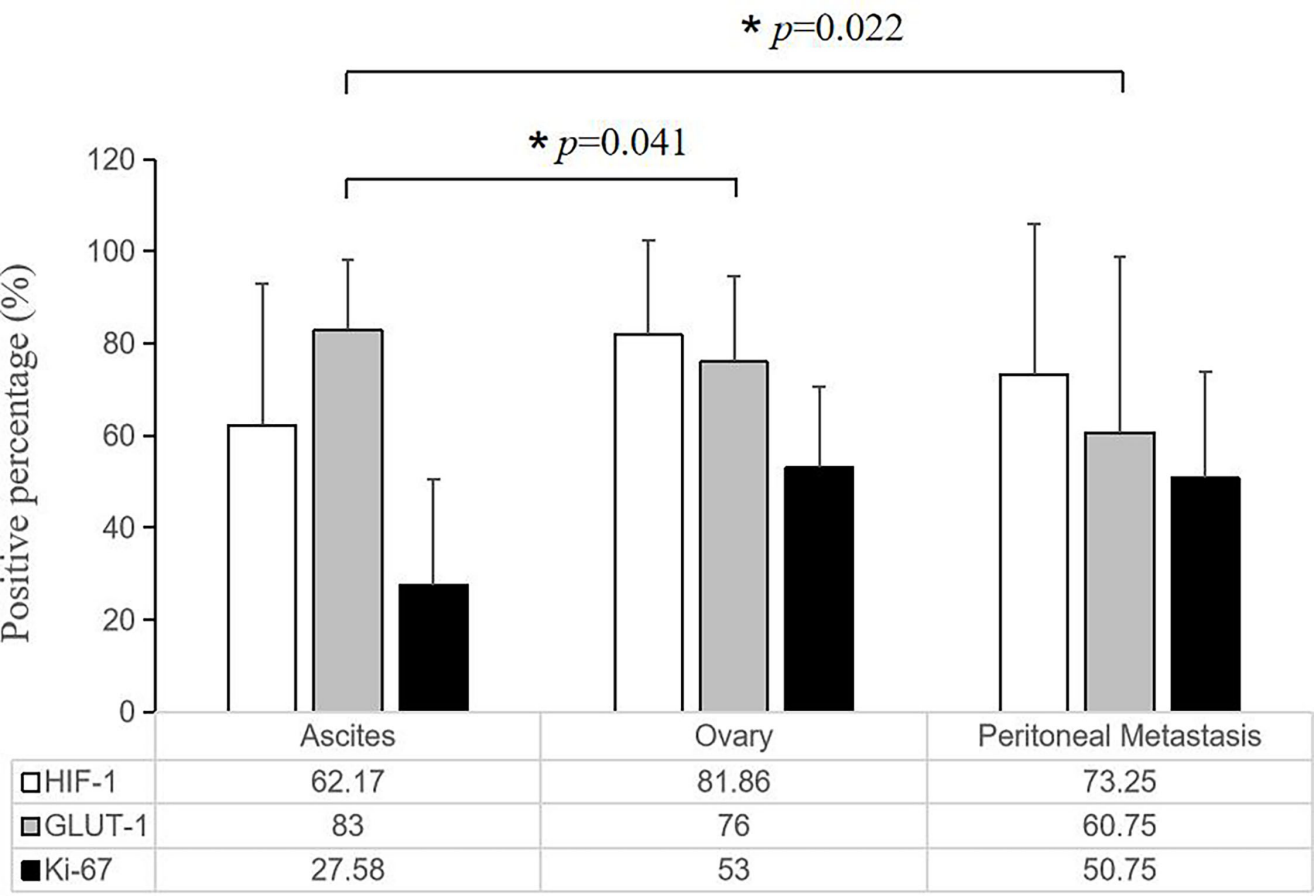
Quantitative comparison of the expression of HIF-1α, GLUT-1 and Ki-67 among tumor cells in the ascites with corresponding primary and peritoneal lesions. There is no significant difference in the expression of HIF-1α and GLUT-1, but the ascites tumor cells had a relatively low Ki-67 expression level compared with their corresponding primary and solid metastatic lesions (*P < 0.05, the error bars was standard deviation.).

Tumor cells floating in effusions can be scattered or form glands, papillae or cell masses. The maximum diameter is usually no more than 300 μm, most are between 50-150μm. HIF-1α and GLUT-1 were expressed by tumor cells in nearly all cytological cases, regardless of tumor type, although to different extents. In general, the expression level of Ki-67 was low (**Table 2**, **Figure 3**). But for the corresponding histological specimens, the expression of these antibodies has significant heterogeneity (**Figure S1**). There is no significant difference in the expression of HIF-1α and GLUT-1 between effusion tumors and their primary or metastatic lesions. But for Ki-67 index, we found that tumor cells in ascites had a relatively low expression level compared with their corresponding primary or metastatic lesions which was statistically significant (*P*<0.05) (**Figures 4**, **5**).

**Table 2:** Immunohistochemical results of tumor cells in body cavity effusions.

Case No.	HIF-1α	GLUT-1	Ki-67
	Percentage of positive cells (%, mean ± SD)		
1	62 ± 6.04	82.6 ± 6.69	24.2 ± 5.50
2	19.4 ± 4.04	100 ± 0	26.6 ± 2.70
3	99.6 ± 0.89	100 ± 0	72.6 ± 10.41
4	88.2 ± 10.64	100 ± 0	77.8 ± 7.40
5	91± 8.49	100 ± 0	90.4 ± 2.70
6	65.2 ± 8.14	52.6 ± 6.50	4.6 ± 2.61
7	69.4 ± 9.34	77.4 ± 6.73	21.6 ± 7.23
8	82.2 ± 5.54	92.8 ± 5.26	91.8 ± 4.44
9	16.2 ± 2.78	100 ± 0	47.8 ± 8.76
10	70.4 ± 7.09	62.8 ± 7.23	11.6 ± 3.78
11	94.8 ± 7.95	76.6 ± 9.15	24.4 ± 7.30
12	61.4 ± 7.96	50.4 ± 8.79	79.6 ± 7.54
13	72.4 ± 9.26	96.8 ± 6.10	17.8 ± 4.60
14	62.2 ± 7.66	63.4 ± 8.33	31.2 ± 8.35
15	79 ± 5.87	95 ± 4.12	85.8 ± 6.53
16	44.6 ± 8.47	63.2 ± 10.33	42.4 ± 7.93
17	91.2 ± 6.30	74.2 ± 5.12	26.4 ± 5.32
18	33.8 ± 7.92	5.8 ± 3.35	81.6 ± 8.14
19	93.2 ± 6.69	81.2 ± 12.87	51.6 ± 10.02
20	22.4 ± 7.06	96 ± 5.66	5.4 ± 1.52
21	4.8 ± 1.64	91.6 ± 7.37	39.4 ± 10.57
22	94.6 ± 8.08	82 ± 7.58	62.8 ± 8.41
23	43 ± 10.00	76.4 ± 6.03	3.4 ± 2.30
24	86.8 ± 9.34	83 ± 7.35	17.4 ± 5.41
25	8.8 ± 3.96	61.8 ± 7.33	12.4 ± 3.21
26	69.2 ± 8.17	0	10.6 ± 4.67
27	63.4 ± 13.01	72.4 ± 5.32	21.6 ± 7.27
28	42.2 ± 6.61	63 ± 8.12	4.2 ± 2.39

**Figure 6:**
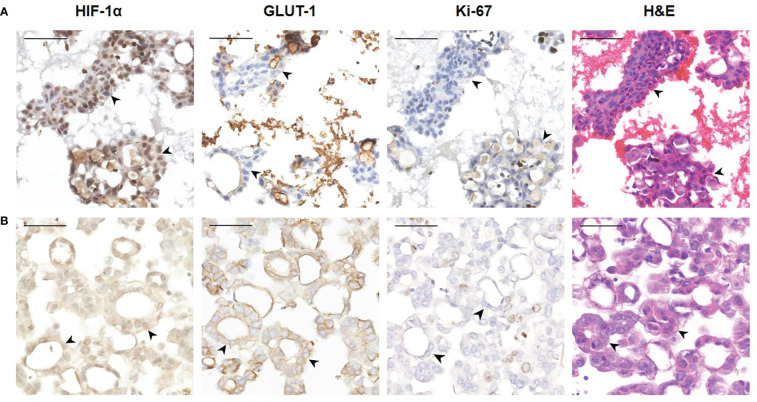
Representative images showing HIF-1α, GLUT-1 and Ki-67 expression of human tumor cells in pleural effusion. **(A)** A 63-year-old female was diagnosed with adenocarcinoma of the lung. The black arrowheads show clusters of tumor cells in pleural effusion (400×). These tumor cells are HIF-1a positive and GLUT-1 negative with a low Ki-67 index. *Bar*, 50 μm. **(B)** A 72-year-old female was diagnosed with breast carcinoma. The black arrowheads show a glandular arrangement of tumor cells in pleural effusion (400×). These tumor cells are HIF-1a positive and GLUT-1 positive with a low Ki-67 index. *Bar*, 50 μm.

**7** cytological cases of pleural effusion were also included in the current study, including 6 cases of lung adenocarcinoma and 1 case of breast carcinoma. Based on the similar characteristics of pleural and peritoneal cavity, tumor cells in pleural effusion were also positive for HIF-1α and GLUT-1 and had a relatively low Ki-67 index (**Figure 6**). Due to the limited number of cases, statistical analysis is not available.

#### 3.2.1 Human malignant ascites and pleural effusion showed high uptake of ^18^F-FDG

**Figure 7:**
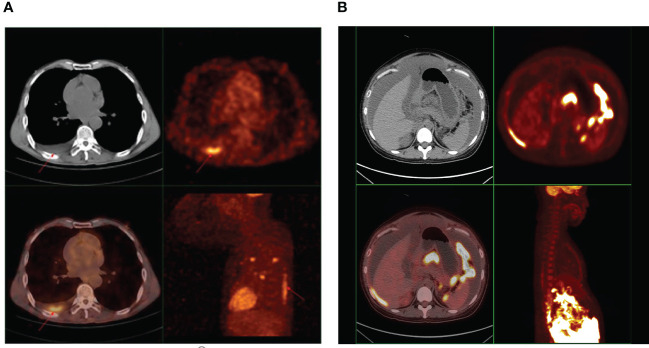
**(A)** A representative PET image showing ^18^F-FDG uptake in pleural effusion of cancer patients. A 58-year-old male diagnosed with adenocarcinoma of the lung, underwent whole-body PET/CT examination. The image showed ^18^F-FDG hypermetabolism in pleural effusion, and the SUV_max_ was 6.9. **(B)** A representative PET image showing ^18^F-FDG uptake in ascites of cancer patients. A 27-year-old female diagnosed with high-grade serous carcinoma of the ovary, underwent whole-body PET/CT examination. The image showed ^18^F-FDG hypermetabolism in ascites, and the SUV_max_ was 2.18.

Among the 28 cases we collected, 13 cases received PET/CT imaging. We collected their PET/CT images and measured the SUVmax value of effusions. We found that ^18^F-FDG uptake of effusions in 9 cases was increased to varying degrees (**Table 3**, **Figure 7**). SUVmax ranged from 2.18 to 6.9.

**Table 3:** ^18^F-FDG uptake of effusion tumors.

No.	Primary site	SUVmax of effusion tumors
1	Ovary	2.86
2	Ovary	/
3	Ovary	3.71
4	Lung	5.97
5	Ovary	/
6	Ovary	2.18
7	Stomach	/
8	Ovary	3.21
9	Ovary	3.41
10	Lung	6.9
11	Ovary	5.52
12	Lung	3.48
13	Ovary	/

## 4 Discussion

Malignant ascites is a common complication of abdominal and pelvic cancer, such as ovary carcinoma, gastrointestinal carcinoma, often indicating that malignant tumors have reached an advanced stage and thus leading to a shorter survival and lower quality of life (29, 30). During the past two decades, the 5-year survival rate of advanced cancer patients with malignant ascites has barely improved (12, 13), which suggests that the current treatments cannot effectively remove malignant ascites. Therefore, it is urgent to further clarify the biological characteristics of ascites tumors in order to seek better targeted therapeutics.

The purpose of this study is to observe the hypoxia and proliferation status of tumor cells floating in ascites in tumor bearing mice as well as in cancer patients. Our previous studies on the animal models of disseminated peritoneal disease have showed that microscopic disseminated peritoneal tumors were intensely hypoxic (16–20). Given that tumor hypoxia is a driving factor for radiotherapy and chemotherapy resistance, if the pattern of tumor hypoxia in ascties tumors is similar with disseminated peritoneal disease, the therapeutic efficacy of current treatments could be compromised.

In our previous animal studies, we used colorectal cancer and lung cancer cell lines to make tumor bearing models, and found that peritoneal micro-metastases were highly hypoxic (16, 19). However, when we collected human ascites samples, we found that ovarian cancer was the most common malignant effusions in clinic. Therefore, the cases we collected were mainly ovarian cancer, and also included a variety of tumor types, including colorectal cancer, gastric cancer, breast cancer and lung cancer. Our current and previous studies have shown that no matter what type of tumor, they all share similar biological characteristics. Tumor cells in effusions and micro-metastasis are in a state of high hypoxia and low proliferation, which was tumor-type independent.

In the established animal model, we performed exogenous hypoxia and proliferation marker injection and autoradiography to observe the hypoxia pattern of tumor cells floating in ascites. But those techniques cannot be applied to human specimens. Pimonidazole is a 2-nitroimidazole compound that is selectively reduced and binds to intracellular macromolecules in hypoxic regions of tumors. It has been widely used in animal experimental studies of tumor hypoxia, but it has not been approved by FDA for human patients, nor dose it apply to retrospective studies. Therefore, we used the method of immunohistochemical staining of endogenous hypoxia factor hypoxia-inducible factor-1 alpha (HIF-1α), Glucose Transporter-1 (GLUT-1) and proliferation marker Ki-67. Although there are certain limitations, this is the simplest and most straightforward technique currently applicable. As a pilot study, we intend to explore the hypoxic characteristics of human ascites tumors through feasible methods, so as to provide basis for further research.

PET imaging with ^18^F-FDG has emerged as an important clinical tool for cancer detection, staging, and monitoring of response and is routinely used in the clinic (31). The uptake of ^18^F-FDG, an analog of glucose, is largely proportional to the rate of glucose metabolism, enabling this parameter to be quantified (32). In hypoxic conditions, cancer cells may undergo a switch from aerobic to anaerobic glucose metabolism. This adaptive response involves the coordinated expression of many HIF-regulated proteins, such as GLUT-1, and various glycolytic enzymes (33). Therefore, the uptake of ^18^F-FDG can reflect the hypoxic state of tumor cells to a certain extent. Moreover, in our previous study, it was found that the uptake of ^18^F-FDG by hypoxic tumor cells was highly correlated with the uptake of hypoxia probe ^18^F-FMISO and pimonidazole (19). Given that ^18^F-FMISO was not routinely used for clinical examination of patients in our institution, ^18^F-FDG imaging was used instead to indirectly reflect the hypoxic state of tumor cells.

Our detection revealed that ascites of tumor bearing mice showed high uptake of ^18^F-FDG and were strongly positive for hypoxia marker pimonidazole and GLUT-1. The results of these animal studies suggested that ascites tumors are hypoxic.

We collected PET/CT images of some cancer patients with malignant effusions and found that malignant ascites and pleural effusion showed high ^18^F-FDG uptake, which could also reflect the hypoxic state of tumor cells floating in effusions.

Studies on human cytological specimens revealed that HIF-1α and GLUT-1 were expressed by tumor cells in nearly all cytological cases of ascites, although to different extents, which indicated that human ascites tumors were also in hypoxic state, which is consistent with the results of animal models. The solid tumor cells located in the primary or metastatic lesions have different degrees of blood supply, and their hypoxia and proliferation status are extremely heterogeneous. We found no significant difference in the expression of HIF-1α and GLUT-1 between ascites tumors and their primary or metastatic lesions and HIF-1α expression in ascites tumors was even lower than that in primary or metastatic lesions numerically. The reason may be related to the fixation time of the specimen. Histological specimens can be fixed immediately once *in vitro*, while cytological specimens are usually exposed to air for a long time before fixation due to the preparation process, which may lead to the changes of the hypoxia state in tumor cells. Based on these factors, HIF-1α expression in ascites tumors may be underestimated.

For cell proliferation, we found that most cases had a relatively low Ki-67 index, which was in sharp contrast to their corresponding primary or metastatic lesions. Our previous experiments have confirmed that ascites is an extremely hypoxic environment (20). The hypoxic tumor cells floating in it have no blood supply and can only survive on the glucose in ascites. And published evidence of cellular and animal experiments have showed that these hypoxic tumor cells are quiescent and divide slowly (34–37). Our current results in human specimens confirmed this point of view.

Tumor cells in pleural effusion are also included in the current study and the results are similar with those in ascites, which indicates that as an unique subgroup, tumor cells in effusions share common biological characteristics in hypoxia and are independent of tumor type. Studies involving larger sample size are still needed to further verify.

Based on our findings, human tumor cells in effusions shared a same pattern of hypoxia with that observed in animal experiments, and they were in a state of low proliferation. Therefore, this unique subgroup of tumor cells is very likely to survive traditional radiotherapy and chemotherapy and maybe the key to the treatment of advanced cancer. While the effectiveness of current treatment schemes for these cells is questionable, which needs to be confirmed by further pharmaceutical studies. At the same time, for this special cell population, there is an urgent need for a more effective hypoxia-targeted therapeutic regimen, such as the application of hypoxia-activated prodrugs (HAPs) (38, 39), and researches in this area are also ongoing.

## Data availability statement

The raw data supporting the conclusions of this article will be made available by the authors, without undue reservation.

## Ethics statement

The studies involving human participants were reviewed and approved by the Ethics Committee of Shengjing Hospital of China Medical University. Written informed consent for participation was not required for this study in accordance with the national legislation and the institutional requirements. The animal study was reviewed and approved by Institutional Animal Ethics Committee.

## Author contributions

YuL was responsible for the research design and the implementation of the main experiment; LZ participated in animal experiments and provided technical support; YH participated in human specimen experiments and provided technical support; XY authorized the use of the case retrieval system and data transferring; YoL participated in PET/CT image collection and processing; HX and X-FL contributed to write and revise the manuscript. All authors contributed to the article and approved the submitted version.

## Supplementary material

Supplementary Figure 1 Heterogeneity of the expression of HIF-1α, GLUT-1, and Ki-67 in human ovarian cancer. A 60-year-old female was diagnosed with high-grade serous carcinoma of the ovary. **(A)**. Histological sections of the ovarian primary (4×). Bar, 5 mm. According to the expression of HIF-1α and GLUT-1, we find three different areas. The red square represents a severely hypoxic area; The blue square represents a moderately hypoxic area; the black square represents a mildly hypoxic area. **(B)**. HIF-1α and GLUT-1 are highly expressed in the severely hypoxic area (red square), with a relatively low Ki-67 index (200×). There are a few CD31-positive blood vessels seen in this area. Bar, 100 μm. **(C)**. In the moderately hypoxic area (blue square), the HIF-1α expression is decreased and the Ki-67 index is much higher (200×). One blood vessel can be seen in this field (black arrow head). Bar, 100 μm. **(D)**. In the mildly hypoxic area (black square), HIF-1α is very low and GLUT-1 expression is also low, while the Ki-67 index is high (200×). The vascular density is increased prominently in this area (black arrowheads). Bar, 100 μm.
