# Supplementary figures and images for: Visualization of hypoxia in cancer cells from effusions in animals and cancer patients
Source: Front Oncol. 2022 Dec 22;12:1019360. doi: 10.3389/fonc.2022.1019360 (PMC9820139; doi:10.3389/fonc.2022.1019360)

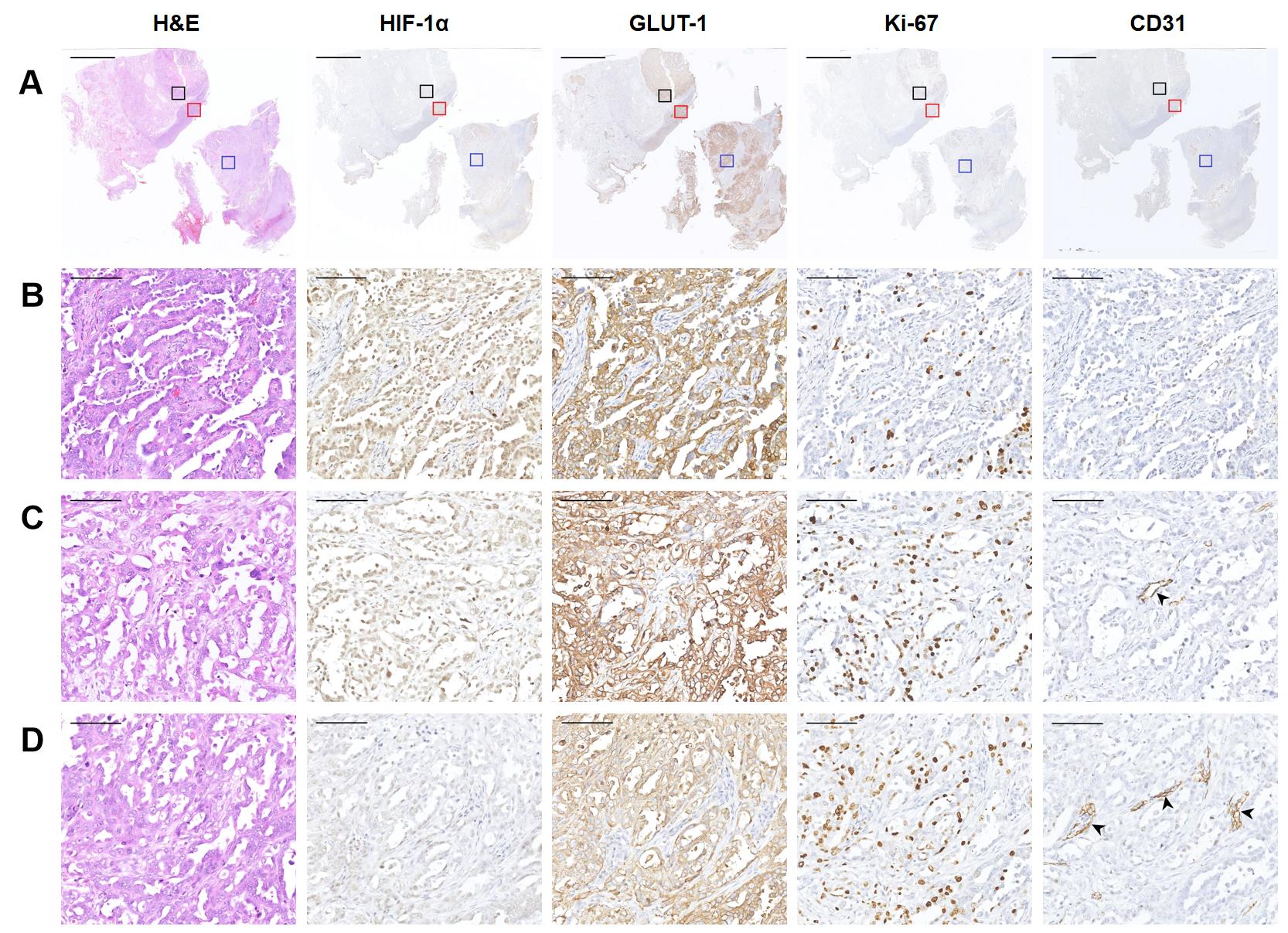

Supplement: Supplementary Figure 1 — Heterogeneity of the expression of HIF-1α, GLUT-1, and Ki-67 in human ovarian cancer. A 60-year-old female was diagnosed with high-grade serous carcinoma of the ovary. (A). Histological sections of the ovarian primary (4×). Bar, 5 mm. According to the expression of HIF-1α and GLUT-1, we find three different areas. The red square represents a severely hypoxic area; The blue square represents a moderately hypoxic area; the black square represents a mildly hypoxic area. (B). HIF-1α and GLUT-1 are highly expressed in the severely hypoxic area (red square), with a relatively low Ki-67 index (200×). There are a few CD31-positive blood vessels seen in this area. Bar, 100 μm. (C). In the moderately hypoxic area (blue square), the HIF-1α expression is decreased and the Ki-67 index is much higher (200×). One blood vessel can be seen in this field (black arrow head). Bar, 100 μm. (D). In the mildly hypoxic area (black square), HIF-1α is very low and GLUT-1 expression is also low, while the Ki-67 index is high (200×). The vascular density is increased prominently in this area (black arrowheads). Bar, 100 μm. [file Image_1.jpeg]
